# Quantification of influenza virus mini viral RNAs using Cas13

**DOI:** 10.1261/rna.080174.124

**Published:** 2025-01

**Authors:** Caitlin H. Lamb, Emmanuelle M. Pitré, Sean Ajufo, Charlotte V. Rigby, Karishma Bisht, Michael S. Oade, Hamid Jalal, Cameron Myhrvold, Aartjan J.W. te Velthuis

**Affiliations:** 1 Department of Molecular Biology, Princeton University, Princeton, New Jersey 08544, USA; 2 University of Cambridge, Department of Pathology, Addenbrooke's Hospital, Cambridge CB2 2QQ, United Kingdom; 3 Public Health England, Addenbrooke's Hospital, Cambridge CB2 2QQ, United Kingdom; 4 Department of Chemical and Biological Engineering, Princeton University, Princeton, New Jersey 08544, USA; 5 Omenn-Darling Bioengineering Institute, Princeton University, Princeton, New Jersey 08544, USA; 6 Department of Chemistry, Princeton University, Princeton, New Jersey 08544, USA

**Keywords:** influenza virus, mini viral RNA, RNA polymerase, Cas13, CRISPR

## Abstract

Influenza A virus (IAV) RNA synthesis produces full-length and deletion-containing RNA molecules, which include defective viral genomes (DVG) and mini viral RNAs (mvRNA). Sequencing approaches have shown that DVG and mvRNA species may be present during infection, and that they can vary in size, segment origin, and sequence. Moreover, a subset of aberrant RNA molecules can bind and activate host–pathogen receptor retinoic acid-inducible gene I (RIG-I), leading to innate immune signaling and the expression of type I and III interferons. Measuring the kinetics and distribution of these immunostimulatory aberrant RNA sequences is important for understanding their function in IAV infection. Here, we explored if IAV mvRNA molecules can be detected and quantified using amplification-free, CRISPR-LbuCas13a-based detection. We show that CRISPR-LbuCas13a can be used to measure the copy numbers of specific mvRNAs in samples from infected tissue culture cells. However, to efficiently detect mvRNAs in other samples, promiscuous CRISPR guide RNAs are required that activate LbuCas13a in the presence of multiple mvRNA sequences. One crRNA was able to detect full-length IAV segment 5 without amplification, allowing it to be used for general IAV infection detection nasopharyngeal swabs. Using CRISPR-LbuCas13a, we confirm that mvRNAs are present in ferret upper and lower respiratory tract tissue, as well as clinical nasopharyngeal swab extracts of hospitalized patients. Overall, CRISPR-LbuCas13a-based RNA detection is a useful tool for studying deletion-containing viral RNAs, and it complements existing amplification-based approaches.

## INTRODUCTION

**FIGURE 1.**
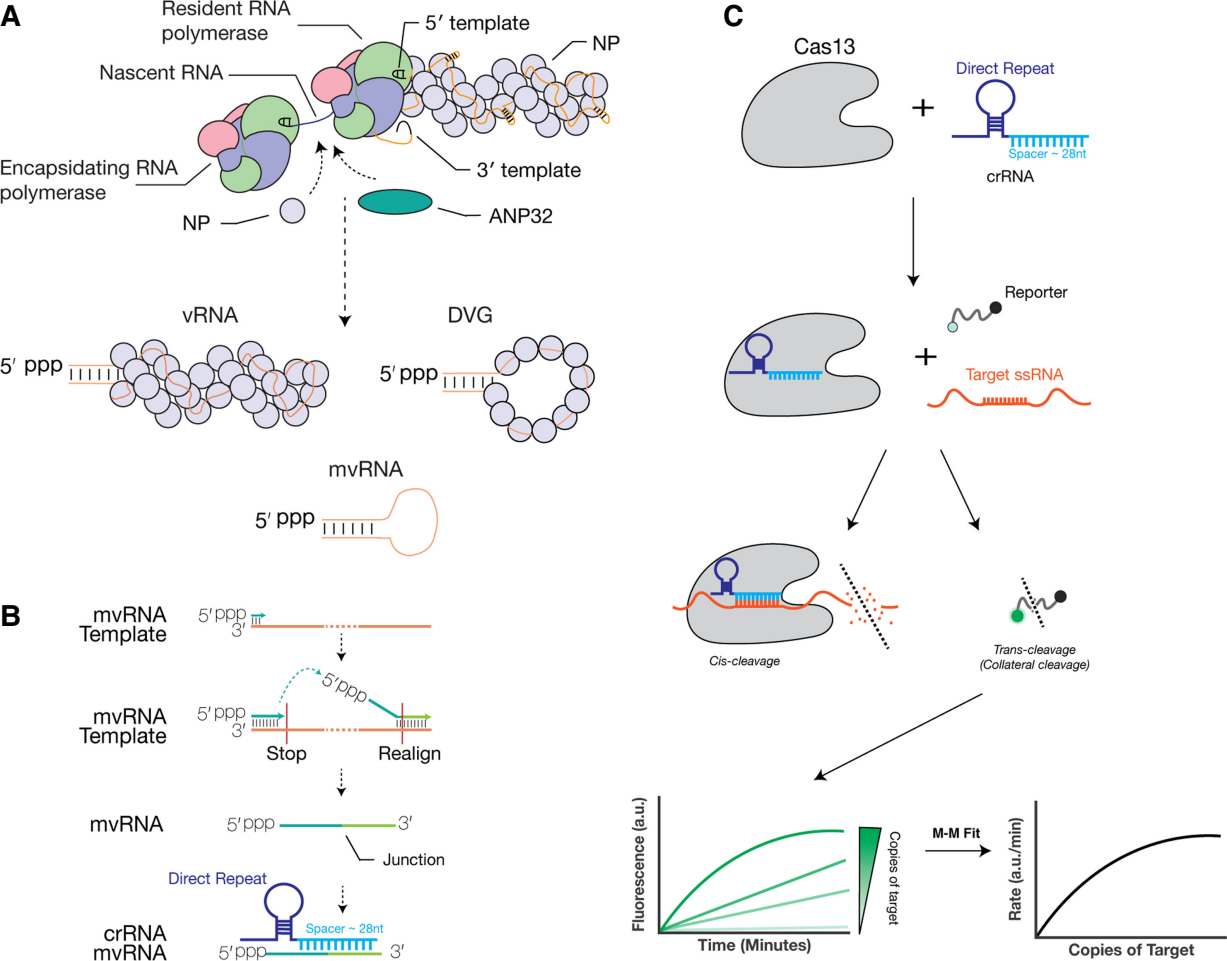
Schematic of IAV RNA replication products and detection mvRNAs using Cas13. (*A*) The IAV RNA polymerase forms a dimer during replication. This dimer is stabilized by host factor ANP32A/B and able to recruit a vRNP to encapsidate nascent viral RNA. In addition to producing full-length RNA products, IAV replication also produces mvRNAs and DVGs. (*B*) During IAV replication, mvRNAs can be generated through a copy-choice recombination mechanism that deletes internal genome segment sequences. crRNAs were designed to target the unique junction sequences, thereby distinguishing mvRNAs from full-length viral genome segments and other mvRNAs. (*C*) Schematic of RNA detection using Cas13.

Influenza A viruses (IAV) cause mild to severe respiratory disease in humans, depending on the viral strain and activation of the innate immune response. Upon infection, IAV releases eight segments of negative-sense, single-stranded RNA that are organized into viral nucleoprotein (vRNP) complexes. Each vRNP complex consists of a viral RNA (vRNA) that is bound by a helical coil of nucleoproteins (NP) and a copy of the RNA-dependent vRNA polymerase (Fig. 1A; te Velthuis and Fodor 2016; te Velthuis et al. 2021). During replication, the IAV RNA polymerase can produce a wide variety of aberrant or nonstandard RNA products, including defective viral genomes (DVG) and mini viral RNAs (mvRNA), which contain the conserved termini of the viral genome segments but lack internal sequences (Fig. 1A; Davis et al. 1980; Baum et al. 2010; Umbach et al. 2010; te Velthuis et al. 2018a; Alnaji and Brooke 2020). It is currently assumed that the internal deletions are the result of an intramolecular copy-choice recombination event that involves pausing of the RNA polymerase at an unknown signal and realignment of the nascent RNA to a complementary sequence downstream. Deletion of an internal sequence results in the formation of a unique junction sequence relative to the full-length viral genome (Fig. 1B).

Some of the aberrant RNAs have been shown to bind and activate retinoic acid-inducible gene I (RIG-I), leading to downstream innate immune signaling (Liu et al. 2015; te Velthuis et al. 2018a; Mendes and Russell 2021; French et al. 2022; Penn et al. 2022). The production of aberrant RNAs plays a still poorly understood role in viral pathogenicity and the outcome of influenza virus infection (Vasilijevic et al. 2017; te Velthuis et al. 2018a; Alnaji and Brooke 2020; Penn et al. 2022). In the case of mvRNAs, their abundance was associated with the appearance of disease markers in mouse and ferret infections with highly pathogenic IAV strains (te Velthuis et al. 2018a; Penn et al. 2022). However, not all aberrant RNA molecules are able to induce an innate immune response. In IAV, as well as other negative-sense RNA viruses, sequence-specific preferences for RIG-I binding and activation have been observed (Baum et al. 2010; Mercado-López et al. 2013; Xu et al. 2015; French et al. 2022). For IAV, we recently showed that mvRNAs containing a template loop (t-loop), a transient RNA structure that can affect RNA polymerase processivity, are more potent inducers of the innate immune response than mvRNAs without a t-loop in cell culture (French et al. 2022), although the sensitivity to t-loops is IAV RNA polymerase-dependent (Rigby et al. 2023). Given the large variety of aberrant RNA sequences produced during IAV infection and the specific effects of different sequences, it is becoming more important to carefully study the kinetics and impact of aberrant RNA species during infection.

Current tools to detect and quantify aberrant RNAs, such as mvRNAs, include primer extension, reverse transcription quantitative PCR (RT-qPCR), and next-generation sequencing (te Velthuis et al. 2018b). All RT and PCR enzymes can introduce errors, including the introduction of point mutations, insertions, and deletions, which may impact the detection of vRNAs and create new molecules that also deviate from the viral genome sequence. Various approaches have been used to minimize these errors, such as circular sequencing. To investigate if we can complement the above assays and perform aberrant vRNA detection without RT and PCR steps, we explored the use of the RNA-guided nuclease Cas13. Specifically, we wanted to explore if we can use Cas13 for direct and amplification-free detection of aberrant IAV RNAs (Fig. 1C). In theory, without enzymatically manipulation of the genetic material that needs to be detected, a more accurate measurement of the quantity of the genetic material can be obtained.

Cas13 uses a CRISPR RNA (crRNA) that consists of a direct repeat region, which is specific to the Cas13 ortholog, and a spacer region, which is designed to be complementary to the target RNA (Gootenberg et al. 2017; Deng et al. 2023). When CRISPR-Cas13 binds to the target RNA, the nuclease activity of Cas13 is activated. Cas13 can subsequently cleave the target, a process that is called *cis* cleavage, as well as any nearby single-stranded RNA molecules, a process called *trans* cleavage or collateral cleavage (Fig. 1C; Abudayyeh et al. 2016; East-Seletsky et al. 2016). The *trans* cleavage activity can be measured with a reporter RNA molecule containing a fluorophore and a quencher (East-Seletsky et al. 2016), making Cas13 suitable for a wide range of applications, including the detection of genomic vRNA (Wang et al. 2019; Abbott et al. 2020; Ackerman et al. 2020; Fozouni et al. 2021; Arizti-Sanz et al. 2022; Zeng et al. 2022). Additionally, the measured fluorescent signal is proportional to the amount of target RNA present in a sample and the number of target RNA molecules present in a reaction can be calculated using a standard curve specific for the target RNA (Fig. 1C; Ramachandran and Santiago 2021). Previous work has used a Cas13 enzyme derived from *Leptotrichia buccalis* (LbuCas13a), to directly detect λ phage ssRNA, β actin mRNA, and SARS-CoV-2 genomic RNA (East-Seletsky et al. 2016; Fozouni et al. 2021).

Building on this previous work, we here explored whether LbuCas13a can be used to detect and subsequently quantify the expression levels of mvRNAs from IAV segment 5 (encoding the vRNP). We chose these mvRNAs, because they had been characterized previously and their short length (approx. 56–125 nt) facilitated chemical RNA synthesis and thus the possibility to use a well-defined input to validate and compare detection methods. We designed our crRNAs such that they targeted the unique junction sequence formed upon mvRNA production (Fig. 1B). This junction differentiates mvRNAs from genomic RNA and DVGs, as well as different mvRNAs from each other. We tested our assays using fractionated (<200 nt) RNA from cell culture, animal tissue, or clinical nasopharyngeal swabs. We find that while we were able to detect mvRNAs in all sample types, LbuCas13-based detection is subject to a trade-off between sensitivity and specificity. crRNAs with the highest sensitivity were not able to differentiate between different mvRNAs that derive from the same gene segment, while crRNAs that detected a single mvRNA sequence did so with a lower sensitivity. We also identified a crRNA that was also able to detect full-length IAV segment 5 without amplification, allowing it to be used for general IAV infection detection in clinical nasopharyngeal swabs. Overall, these results provide new insight into the limitations of LbuCas13-based detection of vRNA and the presence of small, aberrant vRNAs in IAV infection.

## RESULTS

### Amplification of mvRNAs by RT and PCR introduces errors

Different detection methods can be used to quantify IAV aberrant RNA species, but their errors have not previously been compared directly for mvRNAs. To gain insight into the errors generated by these methods, we compared the ability of RT and PCR to quantify known, chemically synthesized mvRNA sequences. Using two mvRNAs of the same length, but with different internal sequences, we observed a lack of consistency in the ability of three different RT and PCR enzyme combinations to produce a single amplification product (Supplemental Fig. S1A). Additionally, the different RT and PCR enzyme combinations amplified the mvRNAs to different levels even though a fixed amount of the synthetic mvRNAs was provided as input (Supplemental Fig. S1A,B). Thus, while primer extension, PCR, and next-generation sequencing are powerful methods to visualize or discover aberrant IAV RNAs, their use as quantification methods appears limited by the enzymes used for the conversion or amplification of different mvRNA sequences.

### LbuCas13a can be used to detect and quantify mvRNAs without amplification

**FIGURE 2.**
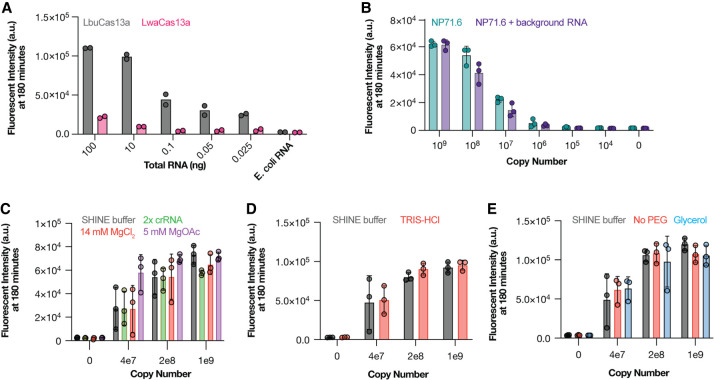
Detection of synthetic mvRNAs using LbuCas13a. (*A*) Detection of 5S rRNA using LbuCas13a or LwaCas13a. (*B*) Detection of synthetic mvRNA NP71.6 diluted in water or HEK293T total RNA using LbuCas13a. Data points indicate technical repeats. (*C*) Comparison of LbuCas13a activity in the standard SHINE buffer (10 nM LbuCas13a, 20 mM HEPES pH 8.0, 60 mM KCl and 5% PEG, 2 U/μL RNase inhibitor [New England Biolabs], 0.25 μM 6UFAM [Supplemental Table S3], 14 mM MgOAc, 5 nM crRNA) to the LbuCas13a activity at a twofold higher crRNA concentration (10 nM); a 14 mM MgOAc to 14 mM MgCl_2_ substitution; a 5 mM MgOAc concentration; (*D*) in the presence of TRIS-HCl instead of Hepes buffer; or (*E*) in the absence of 5% PEG or presence of 5% glycerol. For detection in *C*–*E*, we used synthetic mvRNA NP71.6 diluted in water. For all figures, data points indicate technical repeats.

Cas13 can be used to detect SARS-CoV-2 RNA without (isothermal) amplification and T7 transcription steps when the genome is targeted by multiple guides (East-Seletsky et al. 2016; Fozouni et al. 2021). As only one unique junction is available in mvRNA sequences (Fig. 1B,C), the previously described approach is not feasible for IAV aberrant RNA molecules. However, Cas13 orthologs have variable amounts of *trans*-cleavage activity (East-Seletsky et al. 2016), and this activity can be used to generate additional signal. To determine if Cas13 could potentially be used to detect mvRNA junctions without requiring amplification or T7 transcription steps, we first compared the ability of two Cas13 orthologs, LbuCas13a and LwaCas13a, to directly detect human 5S rRNA in a dilution series of total RNA extracted from HEK293T cells. We observed that LbuCas13a was able to detect 5S rRNA with as little as 0.025 ng of total RNA input, whereas LwaCas13 only produced a signal above background when incubated with 10 ng of total RNA input (Fig. 2A).

We next investigated whether LbuCas13a could detect a previously described, engineered mvRNA based on segment 5 (NP71.6). To detect NP71.6, we designed a crRNA with a spacer sequence complementary to the unique junction sequence relative to the full-length segment 5 vRNA. We subsequently diluted chemically synthesized NP71.6 in water or 100 ng of total HEK293T RNA and found that we were able to detect the mvRNA with a limit of detection <10^6^ copies (Supplemental Table S2), irrespective of the background used (Fig. 2B). No fluorescent signal was observed in the absence of synthetic NP71.6 (Fig. 2B). For our reactions, we used the previously established LwaCas13a SHINE buffer (see methods) and we confirmed that the crRNA, Mg^2+^, PEG, and glycerol conditions of the SHINE buffer were also optimal for LbuCas13a reactions (Fig. 2C–E). Any buffer component substitutions that we explored did not yield significant changes. Using the same approach, we also found that Cas13 can detect the mvRNAs used in Supplemental Figure S1C.

### LbuCas13a can be used to quantify mvRNAs in total cell RNA

**FIGURE 3.**
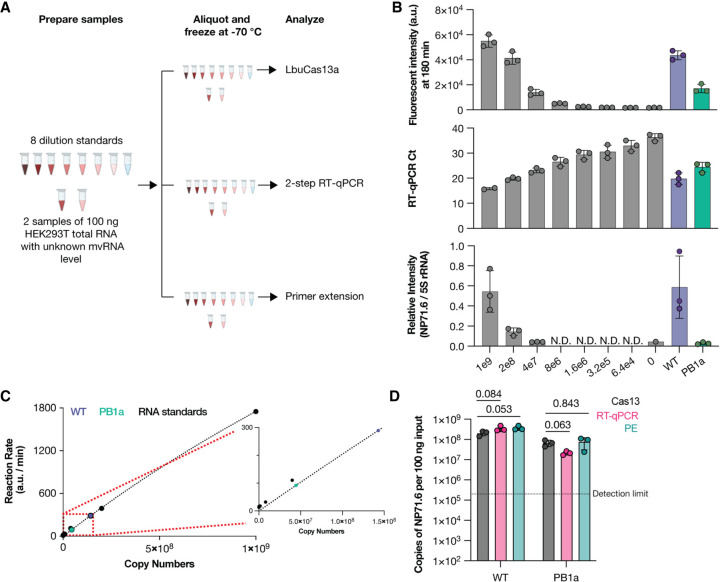
Detection of mvRNAs in cellular RNA samples using LbuCas13a. (*A*) Schematic of RNA sample preparation for mvRNA detection. (*B*) Detection of chemically synthesized (gray bars) or transfected mvRNA NP71.6 in the presence of WT or PB1a mutant IAV RNA polymerase that contained alanine substitutions at positions D445 and D446 (purple or green bar, respectively) using LbuCas13a (*top*), TaqMan-based RT-qPCR (*middle*), or primer extension (*bottom*). Data points indicate three independent repeats. For raw primer extension data, see Supplemental Figure S2. (*C*) Michaelis–Menten fits to the maximum rate of fluorescence as a function of synthetic mvRNA NP71.6 copy number. (*D*) Copy number of mvRNA NP71.6 in transfection samples. Data points indicate three biological repeats. Statistical comparisons are based on nonparametric *t*-test. In all graphs, error bars indicate standard deviation.

We subsequently explored whether we could use LbuCas13a to quantify unknown amounts of NP71.6 in total RNA extracted from HEK293T cells expressing the IAV mini-genome system (te Velthuis et al. 2018b). In this assay, plasmids expressing the three wild-type (WT) IAV RNA polymerase subunits via an RNA polymerase II (Pol II) promoter and the NP71.6 mvRNA via a Pol I promoter were transfected into HEK293T cells. As control, we transfected a PB1 RNA polymerase subunit that contained alanine substitutions at position D445 and D446 (PB1a) instead of the WT PB1 subunit. Total RNA was extracted 48 h posttransfection. To investigate our ability to determine the mvRNA level in HEK293T cells, we first made a fivefold dilution series of chemically synthesized NP71.6 in total RNA from untransfected HEK293T cells. This dilution series was our standard curve (Fig. 3A; Supplemental Fig. S2). Next, we measured the Cas13 fluorescent signal (Fig. 3B), calculated the maximum slope of the fluorescent Cas13 signal for each point of the standard curve, plotted the maximum slope against the known synthetic mvRNA concentrations, and fitted the resulting distribution to the Michaelis–Menten equation (Fig. 3C). Next, the Cas13 signal of the RNA from transfected HEK293T RNA was determined and the fitted standard curve data used to convert the fluorescent signal of the transfected HEK293T cells into mvRNA copy numbers (Fig. 3B,D).

In Supplemental Figure S1, we showed that RT-PCR detection methods can introduce mvRNA amplification biases. To reduce this bias and detect mvRNAs using RT-qPCR in a sequence-specific manner, similar to our Cas13 assay, we introduced a TaqMan probe that was specific for the junction in the NP71.6 mvRNA. When we next compared the quantification obtained using LbuCas13a to the results obtained using primer extension or a two-step TaqMan-based RT-qPCR (Fig. 3B), we observed indeed that our CRISPR-based detection method yielded no significantly different results (Fig. 3D). A similar result was obtained when we expressed the NP71.6 mvRNA in the presence of an inactive IAV RNA polymerase to measure RNA polymerase I (Pol I)-derived input levels (Fig. 3D). Consequently, for a single, well-defined IAV mvRNA the RT-qPCR and Cas13 assays yield comparable results. However, in infection experiments, the mvRNA diversity is relatively large, making it more likely that sequence-based biases arise when amplification steps are used for mvRNA detection.

### Trade-off between LbuCas13a crRNA sensitivity and specificity

**FIGURE 4.**
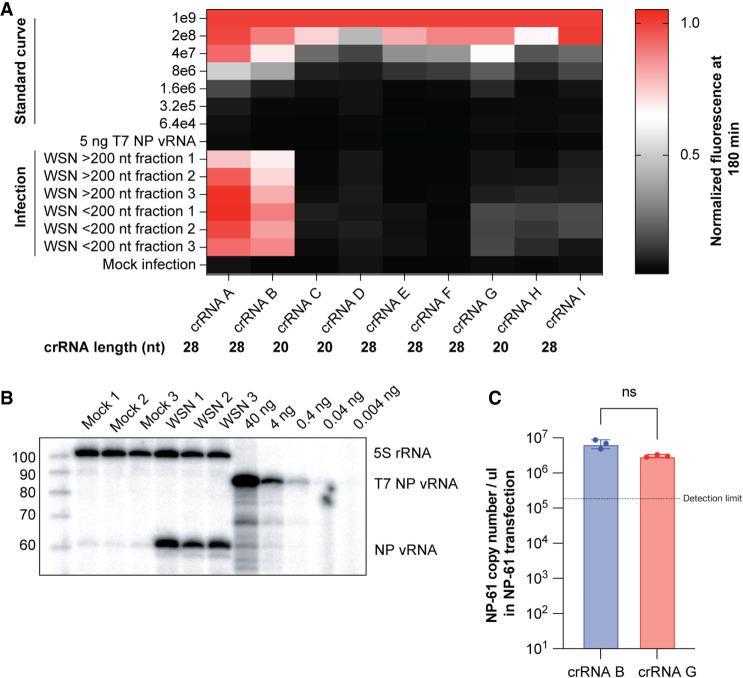
Detection of full-length segment 5 vRNA or NP-61 mvRNA using different crRNAs. (*A*) Heatmap showing normalized fluorescence after 180 min of Cas13 detection. We used nine different crRNA designs (Supplemental Table S2) and tested their ability to detect synthetic NP-61 in standard curve or 5 ng of a T7 segment 5 vRNA transcript in the presence of A549 total RNA. In addition, we fractionated A549 cells infected with WSN into large (>200 nt) and small (<200 nt) RNA fractions. We expected the NP-61 mvRNA to be solely present and detected in the <200 nt fraction. The size of the crRNAs is indicated. (*B*) Primer extension analysis of segment 5 vRNA levels in mock, infected, and T7 segment 5 vRNA transcript samples. The T7 segment 5 vRNA transcript contained a 5′ extension to differentiate it from the WSN segment 5 vRNA. The T7 transcript was diluted in water. (*C*) Copy number of mvRNA NP-61 in HEK 293T transfection. Data points indicate biological repeats of separate transfections.

We next explored whether LbuCas13a could also detect an authentic, conserved, and broadly expressed mvRNA. In addition, we wanted to explore how different crRNAs affect LbuCas13a's ability to detect an mvRNA, and how well a single crRNA can distinguish this NP-61 from other viral sequences, such as the full-length genome segment. We decided to focus on a 61 nt-long segment 5 mvRNA (NP-61). This mvRNA is highly abundant and present in ferret lung tissue infected with A/Brevig Mission/1/1918 (H1N1) or A/Indonesia/2005 (H5N1) or A549 cells infected with A/WSN/1933 (H1N1) (abbreviated as WSN) (te Velthuis et al. 2018a; French et al. 2022). To address the above points, we designed 9 different crRNAs to detect NP-61 with spacer lengths that varied from 20 to 28 nt (Supplemental Table S4). Next, we tested the ability of each cRNA to detect chemically synthesized NP-61, a T7 transcript of full-length segment 5, and vRNA in IAV-infected cells after fractionation into >200 and <200 nt RNA (thereby focusing detection on full-length and DVGs or mvRNAs, respectively). As shown in Figure 4A, we find that the nine crRNAs detected the synthetic NP-61 with different sensitivities and thus limits of detection (LOD), which we determined from the lowest concentration of the standard curve that can be detected 3 SD above the negative control (Supplemental Table S2). The three most sensitive guides were crRNAs A, B, and G (Fig. 4A; Supplemental Table S2). To test which of the crRNAs cross-reacted with full-length segment 5 vRNA, we performed in vitro transcription of segment 5 vRNA. Using primer extension, we determined that ∼5 ng of transcript was representative of the segment 5 vRNA level present in a WSN infection (Fig. 4B). When we subsequently tested the crRNAs against 5 ng transcript in the presence of 50 ng A549 background RNA, we found that none of the crRNAs generated a significant signal (Fig. 4A). We thus conclude that there is none or only limited cross-reactivity with the full-length segment 5 vRNA.

Analysis of fractionated infection samples showed that only guides A and B yielded high signals for the <200 and >200 nt fractions. Since we expected the large fraction to only contain full-length and DVG RNA, and the crRNAs did not support detection of full-length segment 5 vRNA, we suspect that cross-reactivity against segment 5 DVG species had occurred. crRNA G was the only cRNA that yielded a consistent signal above the LOD in all three <200 nt fractions. The signal produced with crRNA G for the <200 nt RNA fraction was lower than the signals obtained with crRNAs A and B. Since the LOD of crRNAs A and B was not substantially different from crRNA G, this result suggests that crRNAs A and B likely trigger cross-reactivity with other mvRNAs derived from segment 5. However, as mvRNAs are diverse in sequence and we cannot chemically synthesize all sequence permutations, we cannot exhaustively test its specificity and must thus assume that crRNA G had limited cross-reactivity with other mvRNAs. To at least confirm that a single authentic mvRNA species can be detected with the same efficiency by cRNAs B and G in a defined context, we transfected NP-61 into HEK 293T cells and extracted total RNA. As shown in Figure 4C, quantification of the NP-61 levels in the transfection sample yielded similar results with the two crRNAs.

Overall, these results suggest that there is likely a trade-off, as in any other nucleic acid detection assay, between crRNA specificity and sensitivity when detecting mvRNAs in complex samples. Since it is impossible to test each guide against all possible segment 5 mvRNAs sequences, we conclude that the detection of a single mvRNA species in a complex sample is likely not possible. In the downstream experiments, we therefore focused on the detection segment 5 mvRNAs with the best sensitivity possible after fractionation to remove any DVG sequences.

### LbuCas13a can detect mvRNAs in RNA extracted from ferret lungs

**FIGURE 5.**
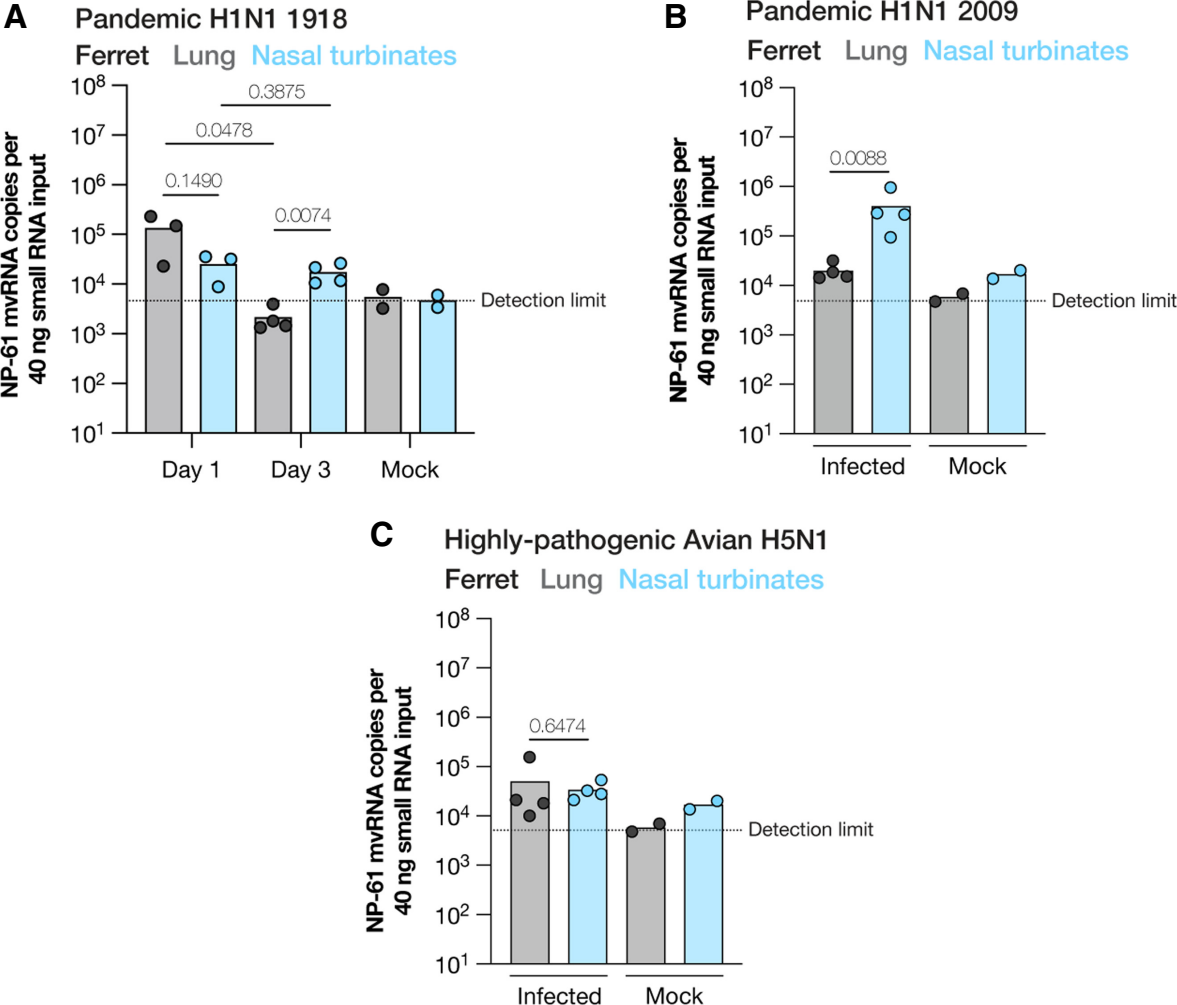
Detection of mvRNAs in ferret lung and nasal turbinates using LbuCas13a. (*A*) Detection of mvRNAs NP-61 in small RNA (<200 nt) fractions extracted from ferret lung or nasal turbinates homogenates infected with influenza A/Brevig Mission/1/1918 (H1N1). (*B*) Detection of mvRNAs NP-61 in small RNA (<200 nt) fractions extracted from ferret lung homogenates infected with influenza A/Netherlands/2009 (H1N1) or (*C*) with influenza A/Indonesia/2005 (H5N1). Data points indicate separate biological infections or mock infections. Statistical comparisons were performed using one-way ANOVA. *P*-values are indicated in the panels.

We had previously shown that mvRNAs can be detected in mouse and ferret lung samples using RT-PCR and next-generation sequencing. We next sought to determine whether we could use Cas13 to detect mvRNAs in RNA extracted from ferret lung or nasal turbinate tissues infected with pandemic A/Brevig Mission/1/1918 (H1N1). Based on the results shown in Figure 4, we suspected that crRNA G would not be sensitive enough to detect mvRNAs. We therefore fractionated the RNA and performed Cas13 detection using crRNA B. As shown in Figure 5A, crRNA B was able to detect mvRNA in the <200 nt sample in ferret lung or nasal turbinate tissues on day 1, and in the nasal turbinates on day 3 postinfection (Fig. 5A). Variation in mvRNA abundance between the infected ferrets was observed. A significant difference in mvRNA abundance was observed between the lung and nasal turbinate homogenates on day 3 (Fig. 5A).

mvRNA sequences vary among IAV strains as the sequences downstream of the promoter are not fully conserved. However, based on our previous deep-sequencing data (French et al. 2022), we found that NP-61 was also produced by the pandemic A/Netherlands/602/2009 (H1N1) and highly pathogenic avian A/Indonesia/5/2005 (H5N1) isolates. We therefore measured the relative abundance of the NP-61 mvRNA and cross-reacting species in ferret lung or nasal turbinate homogenates infected with these two strains. As shown in Figure 5B and C, we were we able to detect mvRNAs in the infected respiratory tract tissues infected by the two IAV strains. A significant difference in mvRNA abundance between the lung or nasal turbinate homogenates was observed in the pandemic H1N1 2009 infections.

### LbuCas13a can detect mvRNAs in RNA extracted from clinical swabs

**FIGURE 6.**
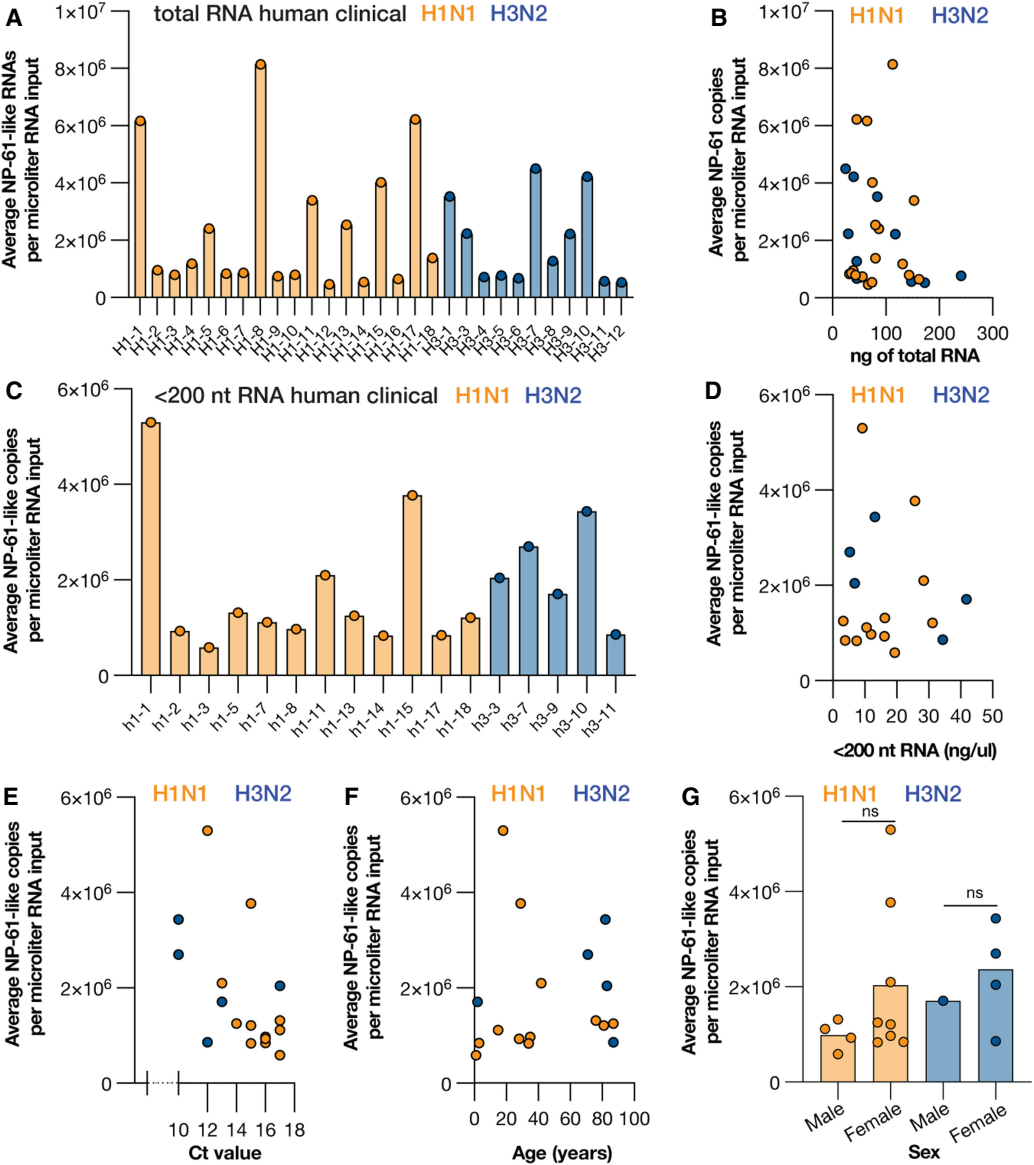
Detection of mvRNAs in total RNA or <200 nt RNA obtained from clinical nasopharyngeal samples using LbuCas13a. (*A*) Copy number of NP-61-like RNAs (mvRNA and DVG) in equal volumes of total RNA extracted from clinical nasopharyngeal samples for the samples with copy number values above the limit of detection. See Supplemental Table S4 for raw values. (*B*) Copy number of NP-61-like RNAs plotted against the amount of ng total RNA used. (*C*) Copy number of NP-61-like mvRNAs in equal volumes of total RNA extracted from clinical nasopharyngeal samples for the samples with copy number values above the limit of detection. See Supplemental Table S5 for raw values. (*D*) Copy number of NP-61-like mvRNAs plotted against the concentration of <200 nt RNA, (*E*) clinical RT-qPCR Ct value, (*F*) patient age, and (*E*) patient sex. Statistical comparisons in *D* are based on nonparametric *t*-test. Not significant is indicated with n.s.

Finally, we investigated whether we could detect mvRNAs in RNA extracted from nasopharyngeal swabs from patients who had been confirmed to be infected with seasonal IAV. The clinical samples obtained were either positive for seasonal H1N1 or H3N2, and were not co-infected with other respiratory pathogens, as determined by clinical RT-qPCR. We first confirmed that we could detect mvRNAs in clinical samples using RT-PCR and found signals corresponding to the expected size in six of 10 IAV-positive clinical samples (Supplemental Fig. S3). Next, we used crRNA B to detect segment 5 RNAs (DVG and mvRNAs) in unfractionated RNA as well as mvRNAs in fractionated RNA. As shown in Supplemental Table S4, we observed positive signals above our limit of detection for 29 of the 30 clinical samples tested when using unfractionated RNA. This suggests that without fractionation, crRNA B could be used for amplification-free detection of IAV infection. Using crRNA B and a synthetic NP-61 standard curve, we converted the signals to number of NP-61-like vRNA copies (i.e., mvRNA and DVG) per µL of extracted clinical sample. This type of copy number was not correlated with the clinical RT-qPCR Ct value (Fig. 6B).

To get an estimate of the NP-61-like mvRNA copies per µl of extracted clinical sample, we performed Cas13-based detection on the fractionated samples. We were able to observe positive signals in fractionated samples for 16 of 17 clinical samples tested (Supplemental Table S5). Using the synthetic NP-61 standard curve, we next calculate the number of NP-61-like mvRNA copies per µl of extracted clinical sample (Fig. 6C). Further analysis showed that these copy numbers were not correlated with the amount of RNA input (Fig. 6D), the reported clinical RT-qPCR Ct value (Fig. 6E), or patient age (Fig. 6F). No significant difference was observed among the H1N1 samples with respect to the reported patient's sex (Fig. 6G). Overall, these findings show that mvRNAs can be detected in a wide range of samples, including influenza virus-positive clinical samples, but quantification of a single mvRNA sequence requires input quantities of RNA that cannot be achieved using animal model or clinical samples.

## DISCUSSION

During IAV infection, aberrant or nonstandard RNA molecules of various lengths and sequences are produced. A subset of these molecules can activate the innate immune response, making them important targets for in-depth characterization. We and others have observed that routinely used RNA quantification methods are sensitive to the vRNA sequence and that they can fail to correctly measure vRNA levels and potentially create additional aberrant signals. We therefore explored the CRISPR-associated enzyme Cas13 as an additional tool to quantify aberrant vRNA levels in cell culture, animal model, and patient samples. Specifically, we focused on the detection and quantification of mvRNAs, which are sufficiently short to synthesize chemically and thus benchmark properly.

In line with previous reports, we showed that the LbuCas13a ortholog can detect and quantify vRNAs without amplification in defined samples that contain a relatively high amount of RNA. We used only a single crRNA, because we specifically wanted to target the unique junctions in an mvRNA. We find that without using multiple crRNAs, the Cas13-based approach can detect a single mvRNA sequence or several closely related sequences if we opt to use a crRNA that has a higher sensitivity. A limitation of the Cas13 approach is the sensitivity to point mutations, in particular when using sensitive crRNAs. This may be an issue for rapidly evolving RNA viruses, which can quickly accumulate point mutations. This issue may be overcome by using occluded Cas13 (Kimchi et al. 2023), which is a new approach that makes Cas13 more sensitive to single nucleotide changes.

For a sample containing a single mvRNA expressed in cells and amplified by the IAV RNA polymerase, LbuCas13a quantifications yielded results that were not significantly different from the primer extension assay (Fig. 3). This result was expected, as any additional aberrant products produced by the IAV RNA polymerase in cell culture (French et al. 2022) were excluded in the PAGE-based size selection that underlies the primer extension analysis. Similarly, the TaqMan-based RT-qPCR produced results that were not significantly different from the Cas13-based assay, because the TaqMan probe hybridized to the mvRNA junction similar to the crRNA of Cas13 and thus also excluded any aberrant PCR product that did not include the junction. For samples where RNA levels are low, a TaqMan-based RT-qPCR assay may be preferable as the LbuCas13 approach currently has a relatively high detection limit. Different combinations of enzymes (Supplemental Fig. S1) can be tested to find the most accurate amplification-based assay. These observations are likely also important for studies of DVG sequences, which also rely on RT-PCR-based sequencing libraries to study sequence variations or identify DVG junctions (Mendes and Russell 2021; Penn et al. 2022).

Using LbuCas13a, we quantified the copy number of a single mvRNA in transfected cells (Fig. 3) and WSN-infected cells (Fig. 4). In infection samples with low RNA concentrations, we could only detect a group of mvRNAs that was related in sequence to a highly abundant mvRNA, because the most sensitive crRNAs have cross-reactivity with other aberrant vRNAs. Although this finding is disappointing and defines a clear limitation for the assay, we were able to detect an mvRNA signal in fractionated clinical samples that were positive for H1N1 and H3N2 seasonal IAV RNA. This result demonstrates that mvRNAs are present in human samples for the first time.

Despite the detection of mvRNAs in clinical samples, we are not able to establish any correlations between mvRNA copy number and clinical Ct value, amount of input material, or patient age and sex (Fig. 6). We expect that any correlations were confounded by unknown clinical factors, as well as when patients were tested in the course of their infection, their immune status, sampling differences, and environmental factors. Other studies have found that influenza-associated morbidity and mortality are often higher in women than men (Serfling et al. 1967; World Health Organization 2008), but we have found no evidence that mvRNAs may underlie this difference.

In summary, understanding the dynamics of IAV aberrant RNAs during infection is important because various lines of research have shown that these RNAs can play a role in the outcome of infection. We here explored the use of Cas13 to detect these IAV RNAs in various sample types. While the assay is fast, amplification-free detection of RNA using Cas13 is subject to a trade-off between sensitivity and specificity, like any nucleic acid detection method. The most sensitive crRNAs detect multiple mvRNAs, even after fractionation, limiting the assay's use for infection samples that have low RNA concentrations. However, when RNA concentrations are sufficient, sequence specificity can be achieved for the highly abundant mvRNA quantified here. We hope that our results will stimulate a more in-depth, dynamic characterization of these IAV nonstandard RNA molecules in order to better understand their role in the outcome of disease.

## MATERIALS AND METHODS

### Preparation of synthetic RNA and primers

Primers were synthesized by Integrated DNA Technologies (IDT) and resuspended in nuclease-free water to 100 μΜ. Primers were stored at −20^o^C and were further diluted prior to analysis. crRNAs were synthesized by IDT and resuspended in nuclease-free water to 100 μM. crRNAs were stored at −70^o^C and were further diluted prior to analysis. Synthetic RNA targets were synthesized by IDT and resuspended in nuclease-free water to 100 μM. Synthetic RNA targets were stored at −70^o^C and were further diluted prior to analysis.

### Plasmids

The pcDNA3-based WSN protein expression plasmids and pPol I-based WSN template RNA expression plasmids were described previously (te Velthuis et al. 2018a; French et al. 2022). The mvRNA-expressed plasmids were generated by site-directed mutagenesis using the primers listed in Supplemental Table S3.

### Cells

HEK293T and A549 cells were originally sourced from the American Type Culture Collection (ATCC). All cells were routinely screened for mycoplasma and grown in Dulbecco's modified Eagle medium (DMEM) containing pyruvate, high glucose, and L-glutamine (GeneDepot) with 10% fetal bovine serum (Gibco). All cells were grown at 37°C and 5% CO_2_.

### Transfections

Transfections of HEK293T cells were performed using Lipofectamine 2000 (Invitrogen) and Opti-MEM (Invitrogen), as described previously (te Velthuis et al. 2018a; French et al. 2022).

### Cell culture infections

Confluent 6-well plates were infected with influenza A/WSN/33 at MOI 3. The cells were incubated with virus inoculum at 4°C for 1 h in DMEM/0.5% FBS to ensure synchronization of the infections. Next, the inoculum was removed, and the cells incubated at 37°C in DMEM/0.5% FBS for 12 h. Mock-infected A549 cells were incubated for 12 h. Cells were lysed in TRI Reagent (Molecular Research Center, Inc.) and subsequently analyzed via RNA extraction.

### RNA sample preparation

RNA extraction was carried out using TRI Reagent (Molecular Research Center, Inc.) following the manufacturer's instructions and as described previously (te Velthuis et al. 2018b). The RNA concentration was determined using a NanoDrop One spectrophotometer (Thermo Fisher) and diluted in RNase-free water prior to analysis.

### RT-qPCR and primer extension

Primers were preannealed to the RNA by heating for 2 min at 95°C prior to an RT step carried out using SuperScript III Reverse Transcriptase (Invitrogen), as described previously (te Velthuis et al. 2018b). After the RT step, probe-based qPCR was carried out using Luna Universal Probe qPCR Master Mix (New England Biolabs) and the QuantStudio3 Real-Time PCR System (Thermo Fisher) using manufacturer's instructions. The primers and probes used are listed in Supplemental Table S3. Primer extensions were performed and analyzed as described previously (te Velthuis et al. 2018a; French et al. 2022).

### Cas13-based detection reactions

Each reaction contained 10 nM LbuCas13a, 20 mM HEPES pH 8.0, 60 mM KCl and 5% PEG, 2U/μL RNase inhibitor murine (New England Biolabs), 0.25 μM 6UFAM (Supplemental Table S3), 14 mM MgOAc, 5 nM crRNA (Supplemental Table S3), and the reported amount of target RNA. Each reaction was first combined in a volume of 44 μL in 96-well plates. After mixing, 20 μL was transferred in duplicate to 384-well plates. The plate was then placed in a BioTek Cytation 5 Cell Imaging Multi-Mode Reader (Agilent) or a BioTek Synergy H1 Plate Reader and incubated at 37°C for 3 h. Fluorescence was measured every 5 min.

### Curve fitting and quantification

The maximum slope of each sample and standard was obtained by first determining the slope of every three data points starting at *t* = 0 min (0–10 min, 5–15 min …170–180 min). The maximum slopes of the standards were then plotted against the known copy numbers of the standards. Standards that reached saturation or quickly reached saturation (slopehigh-concentration << slopelow-concentration) were omitted prior to fitting the curve. The curve was fit to the Michaelis–Menten equation, and *K*_*m*_ and *V*_max_ values were estimated using the Python 3 and the curve-fit trust region reflective (TRF) method from the scipy.optimize package. The *V*_max_ and *K*_*m*_ values were also obtained using Prism 10 software; both Python and Prism 10 software produced the same *V*_max_ and *K*_*m*_ values. Using the obtained *K*_*m*_ and *V*_max_ values and the maximum velocity (*V*), referred to as the reaction rate in the text, of the sample, the unknown concentrations of the samples were estimated. LOD for each crRNA are listed in Supplemental Table S2.

### Clinical samples

Nasopharyngeal samples were taken during routine testing from patients hospitalized at Addenbrookes Hospital, Cambridge, UK, during the 2016–2017, 2017–2018, 2018–2019, and 2019–2020 flu seasons. Patients were positive for either H1N1 or H3N2, but not other respiratory viruses, and samples were taken from a range of pathologies (asymptomatic to death). The study protocol was reviewed and approved by the Health Research Authority (IRAS ID 258438; REC reference 19/EE/0049). Per sample (typically 1.5 mL), 250 µL was used for total RNA extraction using Tri Reagent. RNA was dissolved in 10 µL RNase-free water and stored at −70°C, fractionated by size using the Zymo Research RNA Clean and Concentrator-5, following manufacturer's instruction prior to Cas13 analysis.

### Ferret lung samples

Ferret lung samples were kindly provided by Dr. Emmie de Wit and Dr. Debby van Riel. Ferret experiments were described previously (de Wit et al. 2018), and leftover samples were inactivated and shipped according to standard operating procedures for the removal of specimens from high containment and approved by the Institutional Biosafety Committee. The original experiments were approved by the Institutional Animal Care and Use Committee of Rocky Mountain Laboratories, National Institutes of Health, and conducted in an Association for Assessment and Accreditation of Laboratory Animal Care international-accredited facility, according to the guidelines and basic principles in the United States Public Health Service Policy on Humane Care and Use of Laboratory Animals, and the Guide for the Care and Use of Laboratory Animals.

### Statistical testing

GraphPad Prism10 software was used for statistical testing. Unless otherwise stated, error bars represent standard deviation and individual data points indicate biological repeats.

## SUPPLEMENTAL MATERIAL

Supplemental material is available for this article.

## COMPETING INTEREST STATEMENT

C.M. is a cofounder and consultant to Carver Biosciences and holds equity in the company. The other authors have no conflicts of interest to declare.

## Figures and Tables

**Caitlin Lamb:**
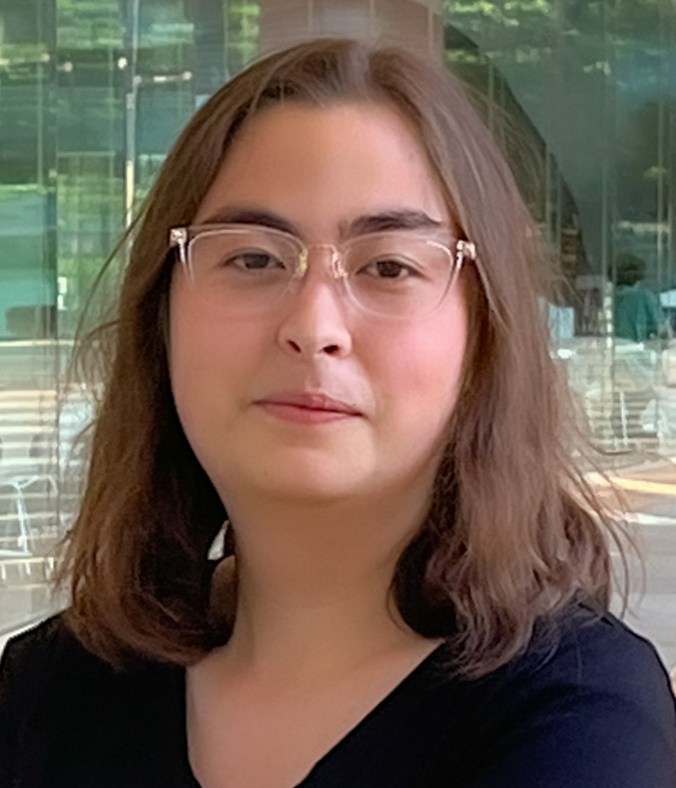


## MEET THE FIRST AUTHOR

***Meet the First Author(s)* is an editorial feature within *RNA*, in which the first author(s) of research-based papers in each issue have the opportunity to introduce themselves and their work to readers of *RNA* and the RNA research community. Caitlin Lamb is the first author of this paper, “Quantification of influenza virus mini viral RNAs using Cas13.” Caitlin is a PhD candidate in the labs of Cameron Myhrvold and AJ te Velthuis at Princeton University. Caitlin's work focuses on understanding the role of small noncanonical viral RNAs in influenza infection and other viruses and detecting these RNAs using CRISPR-Cas13.**

**What are the major results described in your paper and how do they impact this branch of the field?**

Our study aimed to develop an amplification-free approach to study deletion-containing viral RNAs in a sequence-specific manner. Not only were we able to achieve this, but we also underscored the critical role of the CRISPR RNA design, demonstrating trade-offs between sensitivity and selectivity, as observed in other nucleic acid detection methods. This technique can further be adapted to detect and investigate other noncanonical viral and cellular RNAs, such as defective viral genomes and circular RNAs.

**What led you to study RNA or this aspect of RNA science?**

During my first year, I attended faculty talks given by AJ te Velthuis and Cameron Myhrvold. Listening to their talks made me realize the potential of using Cas13 to investigate noncanonical small viral RNAs in influenza virus infection. The mini viral RNAs that I looked at sparked my interest because of the challenges they present in detection and characterization. Studying these influenza virus RNAs deepened my appreciation for RNA biology but also solidified my interest in using innovative molecular tools to further our understanding of mini viral RNAs and their broader implications in infection.

**During the course of these experiments, were there any surprising results or particular difficulties that altered your thinking and subsequent focus?**

We were excited to be able to detect influenza mini viral RNAs in clinical samples, which is something that had not been done before. However, their copy number is not correlated with viral load or patient history, which shows that there are likely a lot of confounding factors that we don't understand yet.

**What are some of the landmark moments that provoked your interest in science or your development as a scientist?**

For my high school chemistry final, my teacher posed a question that still lingers in my mind today: “Why does nature prefer one enantiomer of amino acids over the other?” My tireless attempts to find an answer made me realize that some questions don't have definitive answers—and that's okay. The beauty lies in the process of discovery. This theme of staying curious and forging my own research path has persisted throughout my academic journey. I vividly remember Professor Peter Klopfer encouraging me to keep a notepad to jot down questions and discoveries as I conducted experiments. Professor Michael Boyce pushed me to think more deeply about questions and data, and now, working with Professors Cameron Myhrvold and AJ te Velthuis, I'm encouraged to guide my own research and pursue my interests.

**If you were able to give one piece of advice to your younger self, what would that be?**

The most important advice I can give is to surround yourself with great people. I've been fortunate to have PIs and lab mates who have guided and supported me through research, writing, and personal well-being, and I believe that has made all the difference.
